# Real-world experience with selinexor-containing chemotherapy-free or low-dose chemotherapy regimens for patients with relapsed/refractory acute myeloid leukemia and myeloid sarcoma

**DOI:** 10.3389/fphar.2023.1217701

**Published:** 2023-08-04

**Authors:** Xiwen Tong, Jie Jin, Bin Xu, Shuai Su, Li Li, Mengyuan Li, Yizhou Peng, Xia Mao, Wei Huang, Donghua Zhang

**Affiliations:** Department of Hematology, Tongji Hospital, Tongji Medical College, Huazhong University of Science and Technology, Wuhan, Hubei, China

**Keywords:** acute myeloid leukemia, selinexor, chemotherapy-free, relapsed, refractory

## Abstract

**Introduction:** Treatment of relapsed or refractory acute myeloid leukemia (R/R AML) and myeloid sarcoma (MS) has presented challenges for decades. Studies on selinexor in combination with various standard or intensive chemotherapy regimens for the treatment of R/R AML have demonstrated promising results. This study aimed to evaluate the efficacy and safety of chemotherapy-free or low-dose chemotherapy regimens with selinexor for R/R AML and MS patients.

**Methods:** Ten patients with R/R AML or MS who received chemotherapy-free or low-dose chemotherapy regimens in combination with selinexor at Tongji Hospital from October 2021 to August 2022 were included in this study. The primary endpoint was overall response rate (ORR) and secondary endpoints included complete remission (CR), CR with incomplete hematological recovery (CRi), partial remission (PR), transplantation rate, and safety.

**Results:** All patients were evaluable for response, achieving CR in four (40.0%) patients and CRi in two (20.0%) patients for a total CR/CRi of 60.0%. The ORR was 80.0% when patients with PR were included. Five (50.0%) patients underwent allogeneic hematopoietic stem cell transplantation (allo-HSCT) after treatment with selinexor-containing regimens. At the end of the follow-up, seven (70.0%) patients were alive, and three patients died of transplant-related complications or disease progression. The most frequently reported nonhematologic adverse events (AEs) in patients were grade 1 or 2 asymptomatic hyponatremia.

**Conclusion:** The chemotherapy-free or low-dose chemotherapy regimens in combination with selinexor for R/R AML are feasible and tolerable and provide an opportunity for patients to receive transplantation.

## 1 Introduction

Relapsed or refractory acute myeloid leukemia (R/R AML) has presented a challenge for decades. Despite numerous clinical trials, outcomes are consistently disappointing with 5-year overall survival rates of approximately 10% (DeWolf and Tallman, 2020). The goal of salvage treatment is to achieve a second CR and serve as a bridge to allogeneic hematopoietic stem cell transplantation (allo-HSCT). The most commonly used salvage chemotherapies include FLAG-IDA, MEC, and CLAG with estimated response rates of approximately 40%–50% (Mims and Blum, 2019). However, toxicities such as neutropenic fever, infection, and neurotoxicity are concerns. Chemotherapy-free or low-dose chemotherapy regimens are now recommended for older or unfit patients (DeWolf and Tallman, 2020), but there are few reports in non-elderly patients.

Some patients are candidates for intensive chemotherapy but, for a variety of reasons, may not benefit from it. For example, patients who fail to achieve CR after multiple intensive chemotherapies had a lower probability to achieve CR after another intensive chemotherapy. In addition, some patients have poor bone marrow function after previous chemotherapies and may not tolerate further intensive chemotherapy. Some patients have complications such as infection, poor performance status (PS), or are reluctant to receive intensive chemotherapy, but their disease needs to be controlled as soon as possible. For these patients, the choice of treatment is particularly important because if they can achieve CR again, they have the opportunity for transplant and the potential for longer survival.

Hence, the choice of salvage therapy for the individual patient must include consideration of the associated toxicity profile, as excessive toxicity from the chosen regimen may preclude the feasibility of future stem cell transplantation (Ferrara et al., 2004). Targeted therapies have been shown to be more effective and less toxic than chemotherapy (Isidori and Ferrara, 2021) and the wealth of positive data allows reconsideration of what might soon be new standards of care in younger patients with AML (Kayser and Levis, 2022). Results of several clinical trials confirmed that the early application of targeted agents can result in a deeper and more sustainable remission, with survival benefits for R/R AML patients (Ma and Ge, 2021).

Small molecule inhibitors such as FLT3 inhibitors, IDH inhibitors, and BCL-2 inhibitor as a single agent or in combination with other therapies are new to the arsenal of AML therapy (Rashidi et al., 2018). However, resistance to FLT3 inhibitor, sorafenib and BCL-2 inhibitor, venetoclax has emerged (Gebru and Wang, 2020; Zhang et al., 2022), and some patients have poor tolerance of these agents (Röllig et al., 2015). Second-generation FLT3 inhibitor, gilteritinib trends toward a longer median overall survival effect compared with salvage chemotherapy among patients who received a prior FLT3 inhibitor (Perl et al., 2022), but its high price and medical insurance limit its use. XPO1 is the exclusive, nuclear exporter of most major tumor suppressor proteins and growth regulatory proteins, including p53, p21, p73, FOXO1, and NPM1 (Senapedis et al., 2014; Das et al., 2015; Ishizawa et al., 2015). Higher levels of XPO1 have been independently associated with a worse prognosis in adults with AML (Kojima et al., 2013). Selinexor is an oral, first-in-class selective inhibitor of nuclear export (SINE), specifically blocking XPO1 (Hing et al., 2016). Preclinical studies showed that selinexor has potent cytotoxic activity in AML cell lines and in murine models, including its ability to kill noncycling leukemic stem cells with minimal effects on normal bone marrow (Ranganathan et al., 2012; Etchin et al., 2013; Etchin et al., 2016). Patients with R/R AML received 4, 8, or 10 doses of selinexor in a 21- or 28-day cycle, and 14% of the 81 evaluable patients achieved an objective response and 31% showed ≥50% decrease in bone marrow blasts from baseline (Garzon et al., 2017). There are also other R/R AML trials combining selinexor with intensive chemotherapy (Abboud et al., 2020; Bhatnagar et al., 2020; Fiedler et al., 2020; Martinez Sanchez et al., 2021).

Considering that additional intensive chemotherapy would increase the incidence of complications such as infection, we decided to evaluate chemotherapy-free or low-dose chemotherapy regimens with selinexor to achieve remission. There have been several clinical trials of selinexor alone or in combination with other chemotherapeutic agents for the treatment of AML. However, there are few studies of chemotherapy-free or low-dose chemotherapy regimens including selinexor. Herein we report our experience and results of salvage treatment strategies with selinexor-based regimens for patients with R/R AML.

## 2 Materials and methods

### 2.1 Study population

The patients’ enrollment period was October 2021 to August 2022, and the follow-up deadline was January 2023. The inclusion criteria were as follows: 1) age>18 years and<60 years; 2) R/R AML defined as relapse, failure to achieve complete remission (CR), or CR with incomplete hematologic recovery (CRi) after 2 prior lines of treatment (Döhner et al., 2022); 3) The diagnosis of myeloid sarcoma was based on the World Health Organization (WHO) classification version 2022 of haematolymphoid tumours (Cree, 2022). The exclusion criteria were as follows: 1) AML-M3 diagnosed based on the FAB classification; 2) unstable cardiovascular function, liver dysfunction, severe renal dysfunction.

### 2.2 Treatment schedule

(1) azacitidine 75 mg/m^2^/d on days 1–7, venetoclax dosing began at 100 mg on day 1 and increased stepwise to reach the serum level of 1,000 and 1,500 ng/mL (voriconazole 100 mg/d or 200 mg/d will be used to raise the venetoclax serum level), and we will begin monitoring serum levels of venetoclax after 1 week of treatment; selinexor was initially dosed at 35 mg/m^2^ by mouth administered in 4-week long cycles of twice weekly for 3 weeks with 1 week off to improve tolerability.
(2) homoharringtonine 1 mg/d on days 1–5, bortezomib 1.3 mg/m^2^ on days 1, 4, 8, 11 or once weekly for 4 weeks, G-CSF 300 ug/d, and selinexor (usage is as described above). Minor adjustments were made for each patient, and the regimens are detailed in Table 2.

Bone marrow assessments were performed weekly after treatment. Patients achieving CR or CRi in 2 weeks were recommended for allo-HSCT as soon as possible. Patients with bone marrow blast percentage decrease more than 50% in 2 weeks but not achieving CR after treatment, were continued with selinexor treatment. The change of regimen will be considered when one of the following situations occurs: 1) the patient’s bone marrow blast percentage decreases less than 50% in 2 weeks after treatment; 2) the patients mentioned above still have not achieved CR in 3 weeks.

### 2.3 Safety

Adverse Events (AEs) were classified according to Common Terminology Criteria for Adverse Events (CTCAE V5.0). AEs were classified as severe AEs, drug-related AEs, AEs of special concern (nausea, vomiting, neurological toxicity), and AEs that lead to discontinuation of treatment. The frequency, severity, and causal relationship of AEs were analyzed by the system organ class. To minimize nausea, all patients received 5-HT3 antagonists starting before the first dose of selinexor and continued two to three times a day as needed.

### 2.4 Evaluation and definition

Bone marrow assessments were performed weekly after treatment. As for gastric myeloid sarcoma, ultrasound, CT, and PET-CT were used after treatment. MRD was evaluated according to European LeukemiaNet (ELN) version 2022 (Döhner et al., 2022). Bone marrow aspiration was assessed for MRD using multiparameter flow cytometry combined with real-time quantitative polymerase chain reaction (RT-qPCR) as follows:

1) MRD assessed by multiparameter flow cytometry (MFC): MRD negativity was defined as<0.1%. 2) MRD assessed by RT-qPCR: abnormal genes associated with prognosis were used for molecular MRD detection (Heuser et al., 2021). 3) WT1 was used for molecular MRD assessment if no molecular marker was available at diagnosis, MRD negativity was defined as <0.6% (Wang et al., 2020). Subjects were defined as MRD-negative when MFC and molecular marker/WT1 were both negative in two consecutive samples.

The primary objective was to determine the overall response rate (ORR) and secondary objectives were to determine the complete remission (CR) rate, incomplete hematological recovery (CRi) rate, partial remission (PR) rate, toxicities, and allo-HSCT rate, defined as the number of patients who proceeded to allo-HSCT following remission. CR was defined as being transfusion independent with an absolute neutrophil count (ANC) > 1.0 × 10^9^/L, platelet count >100 × 10^9^/L, bone marrow blasts <5%, absence of circulating blasts, and absence of extramedullary disease. CRi was defined as meeting all CR criteria except for residual neutropenia (<1.0 × 10^9^/L) or thrombocytopenia (<100 × 10^9^/L). PR was defined as a decrease in pre-treatment bone marrow blast percentage by at least 50% and to within the range of 5%–25%, while otherwise meeting all hematologic criteria of CR (Döhner et al., 2022). CR, CRi, and PR were counted toward the ORR.

### 2.5 Statistical analysis

All data were analyzed using SPSS 25.0. Continuous variables were described with the median and interquartile range (IQR) or range. Categorical variables were presented using frequencies and percentages.

## 3 Results

### 3.1 Patient characteristics

Between October 2021 and August 2022, we enrolled nine patients with R/R AML and one patient with gastric myeloid sarcoma, including six (60.0%) males and four (40.0%) females in this study. The follow-up deadline was January 2023. The median age of patients was 46 years (range 33–56 years). Seven (70.0%) patients had an ECOG performance status of 0 or 1 and 3 (30.0%) had an ECOG performance status of 2. In R/R AML patients, five patients relapsed within 12 months, one after 12 months, and three were refractory. According to the 2022 ELN guidelines (Döhner et al., 2022), four (40.0%) and three (30.0%) were classified as adverse, and intermediate genetic risk, respectively. The most frequently mutated genes were RUNX1 (30.0%), MLL (30.0%), FLT3 (30.0%), IDH2 (20.0%), and CEBPA (20.0%). The median bone marrow blast count was 10.25% (IQR 6.13, 47.50) before treatment containing selinexor. And one patient received autologous HSCT before. The median prior lines of chemotherapy that patients received were four (IQR 1, 6), and four out of ten (40.0%) patients had received more than five prior lines of chemotherapy and one patient received up to 13 cycles of chemotherapy. The patient demographics and characteristics are shown in Table 1 (the results of the chromosome karyotype and gene mutation, and regimens that patients received are provided in Table 2).

**TABLE 1:** Baseline data of patients.

Characteristics	Total (*n* = 10)
Age, years, median (range)	46 (33–56)
Patient gender, n (%)	
Male	6 (60.0%)
Female	4 (40.4%)
Type of disease	
AML-M1	1 (10.0%)
AML-M2	5 (50.0%)
AML-M4	2 (20.0%)
AML-M5	1 (10.0%)
Myeloid Sarcoma	1 (10.0%)
ECOG	
0	3 (30.0%)
1	4 (40.0%)
2	3 (30.0%)
Remission duration	
≤12 m	5 (50.0%)
>12 m	1 (10.0%)
Refractory disease	3 (30.0%)
ELN genetic group	
Favorable	1 (10.0%)
Intermediate	3 (30.0%)
Adverse	4 (40.0%)
Unknown	2 (20.0%)
Post-HSCT	1 (10.0%)
Results before treatment	
CBCs, median (IQR)	
WBC(×10^9^/L)	3.54 (0.50–6.16)
ANC (×10^9^/L)	1.64 (0.05–4.59)
Hb (g/L)	87.50 (69.75–95.50)
PLT (×10^9^/L)	32.00 (18.75–178.50)
Bone marrow blasts, %, median (IQR)	10.25 (6.13–47.50)
Extramedullary infiltration, n (%)	1 (10.0%)
Molecular abnormality, n (%)	9 (90.0%)
Chromosome abnormality, n (%)	4 (40.0%)
Previous therapy cycles, median (IQR)	4 (1–6)
At the end of follow-up	
Allo-HSCT	5 (50.0%)
Status of disease	
CR	6 (60.0%)
NR	4 (40.0%)
Survival, n (%)	7 (70.0%)

CBCs, complete blood counts; WBC, white blood cell; ANC, absolute neutrophil count; Hb, hemoglobin; PLT, platelets; CR, complete remission; NR, non-remission; AML, acute myelocytic leukemia; MDS, myelodysplastic syndromes; HSCT, hematopoietic stem cell transplantation; ECOG, eastern cooperative oncology group; ELN, European LeukemiaNet.

**TABLE 2:** The results of the chromosome karyotype, gene mutation and regimen of patients.

No./Diagnosis	Gender/Age	Chromosome karyotype/Gene mutation	Previous therapy cycle	Reasons for choosing this regimen	Treatment	Outcomes after selinexor	HSCT	Status at the follow-up
1	Female	Unknown	0	Gastric lesions are prone to perforation	AZA 75 mg/m^2^/d IH d1-7+ VEN 100 mg qd increased stepwise to reach the dose of 200 mg + selinexor 35 mg/m^2^ biw for 2 weeks	PR	Yes	CR
Gastric myeloid sarcoma	37	Unknown		Acute cholecystitis and percutaneous transhepatic drainage of the gallbladder was made				Survival
2	Male	Unknown	1	Primary resistance	AZA 75 mg/m^2^/d d1-7+VEN 200 mg qd + selinexor 35 mg/m^2^ qw for 3 weeks	CR	Yes	CR
AML-M2	53	IDH2; WT1; PTPN11; U2AF1; MLL-PTD		Gene mutations with poor prognosis		MRD (−)		Dead
3	Male	46, XY	4	Primary resistance	HHRT 1 mg/d d1-5+ bortezomib 1.3 mg/m^2^ biw for 2 weeks + G-CSF 300ug/d IH d1-14+ selinexor 35 mg/m^2^ biw for 2 weeks	CRi	Yes	NR
AML-M5	46	FLT3-TKD; RUNX1; WT1				MRD (−)		Survival
4	Male	47, XY, +8[1]/46, X, -Y [2]/46, XY [7]	13	No response to multiple chemotherapies	AZA 75 mg/m^2^ d1-7+ VEN 100 mg qd + selinexor 35 mg/m^2^ biw for 2 weeks	CR	No	CR
AML-M4	56	ASXL1; DNMT3A; IDH1; IDH2				MRD (−)		Survival
5	Male	46, XY [7], del (3), del (6), t (7;12), +11	2	Primary resistance	Bortezomib 1.3 mg/m^2^ qw for 4 weeks + VEN 200 mg qd + selinexor 35 mg/m^2^ biw for 2 weeks	PR	No	NR
				Gene mutations with poor prognosis				
AML-M2	33	FLT3-ITD; RUNX1; WT1; MLL-PTD		No response to intensive chemotherapy		MRD (+)		Dead
				Perianal infection				
6	Female	46, XX	10	Recurrent perianal infection	AZA 75 mg/m^2^/d d1-7+ chidamide 30 mg biw + bortezomib 1.3 mg/m^2^ qw for 4weeks + selinexor 35 mg/m^2^ biw for 2 weeks	NR	No	NR
AML-M2	39	CEBPA; WT1; KRAS		Bloodstream infection		MRD (+)		Survival
7	Female	46, XX	5	Relapse within 8 months after auto-HSCT	DAC 20 mg/m^2^/d d1-5 + HHRT 2 mg d3-7 + Ara-C 25 mg Q12 h IH d3-9 + G-CSF 300ug qd IH + selinexor 35 mg/m^2^ biw for 2 weeks	CRi	No	CR
AML-M1	46	RUNX1; GATA2				MRD (+)		Survival
8	Male	46, XY, del(11)(q23)	5	No response to multiple chemotherapies	AZA 75 mg/m^2^/d d1-5+VEN 100 mg qd + selinexor 35 mg/m^2^ biw for 2 weeks	CR	Yes	CR
AML-M4	34	KRAS; MLL-ELL; MLL-AF6				MRD (+)		Survival
9	Male	46, XY	3	Relapse	AZA 75 mg/m^2^/d d1-7+ VEN 200 mg qd + selinexor 35 mg/m^2^ biw for 2 weeks	NR	No	NR
AML-M2	51	SMC1A; TET2; CEBPA; CSF3R		The patient requested a non-intensive chemotherapy		MRD (+)		Dead
10	Female	45, X, -X, t (8;21) (q22; q22.1)	1	Capillary leak syndrome during previous chemotherapy	AZA 75 mg/m^2^/d qd IH d1-5+ HHRT 2 mg/d d1-5+ Ara-C 50 mg qd d1-5 + selinexor 35 mg/m^2^ biw for 2 weeks	CR	Yes	CR
AML-M2	46	NRAS; JAK2; FLT3-ITD; RAD21; AML1-ETO		Poor bone marrow function after previous chemotherapy		MRD (−)		Survival

AZA, azacytidine; VEN, venetoclax; DAC, decitabine; Ara-C, cytarabine; ACM, aclacinomycin; HHRT, homoharringtonine.

### 3.2 Efficacy

All patients included in the study were evaluable for response, achieving CR in four patients (40.0%) and CRi in two patients (20.0%) for a total CR/CRi of 60.0%. Four patients with CR or CRi achieved MRD-negativity and the median time to MRD negativity was 12 days after treatment. Two (20.0%) patients achieved PR, resulting in an ORR of 80.0% (Table 3). Four (40.0%) patients who achieved CR/CRi after one cycle of a regimen containing selinexor then received allo-HSCT. And one patient with gastric myeloid sarcoma received allo-HSCT after multiple selinexor-containing regimens.

**TABLE 3:** Outcomes of patients after receiving one cycle of regimen containing selinexor.

Characteristics	Total (*n* = 10)
CR	4 (40.0%)
CRi	2 (20.0%)
CR/CRi	6 (60.0%)
PR	2 (20.0%)
NR	2 (20.0%)
ORR	8 (80.0%)

**FIGURE 1:**
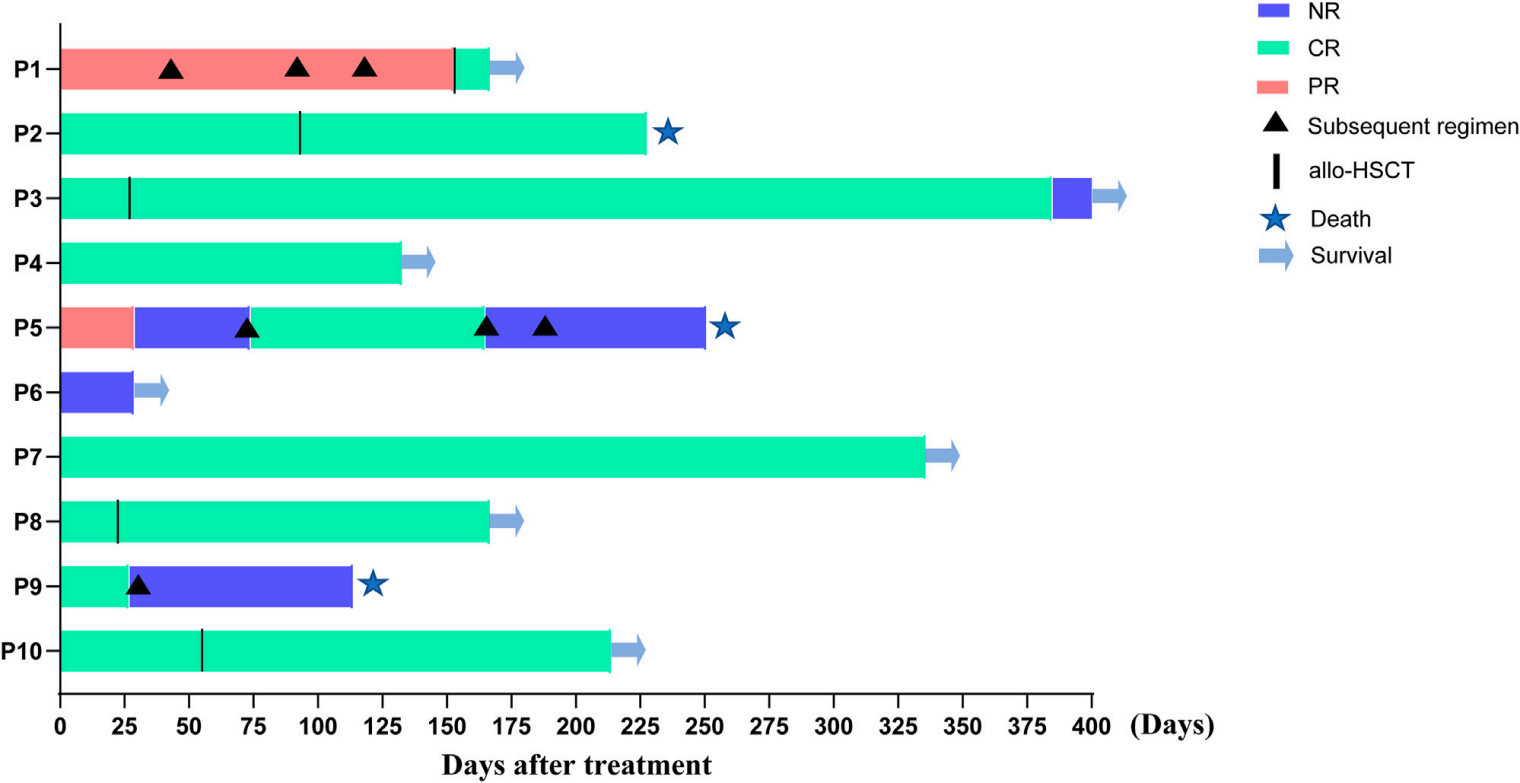
Response to the treatment and overall survival in all the patients included. Note: Patients No.9 did not have regular bone marrow assessment due to personal reasons, so the actual CR period may be shorter than that shown in the figure, hence the curative effect was judged to be NR (shown in Table 2).

At the end of the follow-up, five (50%) patients received allo-HSCT, six (60.0%) patients remained in CR, seven (70.0%) patients were alive, and three patients died of severe transplantation-related complications or disease progression. Four out of five patients who received allo-HSCT were alive at the end of the follow-up (Figure 1).

### 3.3 Safety

All ten patients were included in the safety assessment. As for hematological toxicity, six patients whose time to recovery from neutropenia and thrombocytopenia can be evaluated, the median duration of neutropenia was 7 days (IQR 0,18) and the median duration of thrombocytopenia was 4 days (IQR 0,18) during induction. Of the remaining four patients, two underwent bridge transplantation without neutropenia and thrombocytopenia recovery, one underwent the next chemotherapy treatment before recovery, and the other abandoned treatment and was discharged.

Most of the nonhematologic AEs were mild, eight patients experienced grades 1–2 AEs, and two patients experienced grades 3–4 AEs. One patient suffered acute cerebral infarction and improved after treatment, which was not related to selinexor treatment. The most common nonhematologic AEs occurring were as follows: hyponatremia (100%), nausea (40.0%), fatigue (30.0%) and vomiting (20.0%) (Table 4).

**TABLE 4:** Classification and frequency of total adverse events and according to grade.

Adverse event	Grade 1	Grade 2	Grade 3	Grade 4	Total
Hematologic toxicities					
Anemia	1 (10.0)	3 (30.0)	6 (60.0)	-	10 (100)
Neutropenia	-	-	3 (30.0)	7 (70.0)	10 (100)
Thrombocytopenia	-	-	1 (10.0)	6 (60.0)	7 (70.0)
Leukopenia	-	-	3 (30.0)	7 (70.0)	10 (100)
Febrile neutropenia	-	-	5 (50.0)	-	5 (50.0)
Gastrointestinal disorders					
ALT increased	-	-	1 (10.0)	-	1 (10.0)
AST increased	-	-	1 (10.0)	-	1 (10.0)
Nausea	-	4 (40.0)	-	-	4 (40.0)
Diarrhea	-	-	-	-	0
Hyperbilirubinemia	-	-	-	-	0
Mucositis oral	-	-	-	-	0
Vomiting	-	2 (20.0)	-	-	2 (20.0)
Cholecystitis	-	1 (10.0)	-	-	1 (10.0)
General disorders and administration site conditions					
Fatigue	3 (30.0)	-	-	-	3 (30.0)
Edema limbs	-	-	-	-	0
Weight loss	1 (10.0)	-	-	-	1 (10.0)
Infections					
Sepsis	-	-	-	1 (10.0)	1 (10.0)
Lung infection	-	1 (10.0)	-	-	1 (10.0)
Electrolyte and nutrition disorders					
Hyperglycemia	-	-	-	-	0
Hypokalemia	-	-	1 (10.0)	-	1 (10.0)
Hypomagnesemia	-	-	-	-	0
Hyponatremia	7 (70.0)	3 (30.0)	-	-	10 (100)
Anorexia	-	-	-	-	0
Nervous system disorders					
Headache	-	-	-	-	0
Peripheral motor Neuropathy	-	-	-	-	0
Stroke	-	1 (10.0)	-	-	1 (10.0)

## 4 Discussion

The prognosis of R/R AML and myeloid sarcoma are poor and treatments are challenging. Allo-HSCT is still the most effective treatment for patients who achieve complete remission (CR). Effective salvage chemotherapy regimens are still being explored, how to enable more patients to achieve a second CR and be able to receive transplantation is a challenge.

At present, intensive chemotherapy is still the first choice for patients with R/R AML, but its toxicities and drug resistance provide limited benefits for patients. Considering the development of modes of chemotherapy combined with targeted drugs, chemotherapy-free or low-dose chemotherapy regimens are used in our study. Selinexor is an oral, first-in-class, selective inhibitor of nuclear export compound, which blocks XPO1 function and has shown promising anti-leukemia activity *in vitro* and *in vivo* (Etchin et al., 2016). Studies of selinexor alone or in combination with other chemotherapy drugs have shown good efficacy in the treatment of R/R AML. Selinexor 100 mg/weekly with FLAG-IDA in the treatment of R/R AML achieved a CR/CRi rate of 66.7% (Martinez Sanchez et al., 2021); Selinexor 60 mg on days 1,5,10,12 based on CLAG achieved a CR/CRi rate of 45% in 40 R/R AML patients (Abboud et al., 2020); Selinexor in combination with fludarabine and cytarabine in pediatric R/R AML achieved the CR/CRi rate of 47% in 15 patients (Alexander et al., 2016) and selinexor plus cytarabine and idarubicin in patients with R/R AML achieved a CR/CRi rate of 47.6% (Fiedler et al., 2020). In this study, we proposed chemotherapy-free or low-dose chemotherapy regimens containing selinexor for the treatment of R/R AML and myeloid sarcoma. According to the principle of treatment individualization, factors such as patients’ previous chemotherapy regimens, PS, types of medical insurance, and wishes were considered, so the regimens containing selinexor varied among the ten patients. However, patients in this study achieved an ORR of 80% and a CR/CRi rate of 60.0%, which was similar to or even better than the results reported above. And four patients with CR or CRi achieved MRD-negative remissions.

Of note, we used low-dose chemotherapy or chemotherapy-free regimens with selinexor and achieved results similar to those of selinexor in combination with standard or intensive chemotherapy. These promising results support a new, less intensive treatment option for patients with R/R AML. There are several reasons for our good results: firstly, considering the rapid changes of primary diseases and possible drug resistance in R/R AML patients, bone marrow assessments were performed weekly after treatment to make sure that the treatment will be adjusted promptly, according to the changes in disease; then, the innovative combination of selinexor with venetoclax also enhanced efficacy; at the same time, the treatment regimens containing selinexor were well tolerated by the patients, and there were few cases of treatment interruption or delay due to adverse events.

Nowadays, venetoclax is mainly used in combination with decitabine (DiNardo et al., 2020; Wei et al., 2020; Pollyea et al., 2021), cytarabine (Wei et al., 2019; Karol et al., 2020), and azacitidine (Winters et al., 2019; Pollyea et al., 2021) in the treatment of AML. However, responses to venetoclax correlate closely with the developmental stage, and monocytic AML is more resistant. Mechanistically, resistant monocytic AML has a distinct transcriptomic profile with loss of expression of venetoclax’s target, BCL-2, and reliance on MCL-1 to mediate oxidative phosphorylation and survival (Pei et al., 2020). MCL-1 is a key player in the intrinsic resistance to venetoclax in AML cells (Bogenberger et al., 2014; Niu et al., 2014). Preclinical results showed that selinexor induces responses at well-tolerated doses (Etchin et al., 2016) and decreases MCL-1 protein levels (Lapalombella et al., 2012). Selinexor in combination with venetoclax, modulates MCL-1, which plays an important role in the antileukemic activity of the combination (Luedtke et al., 2018). Fischer, M.A. also found that venetoclax response is enhanced by SINE compounds (Fischer et al., 2020). There are three ongoing clinical trials of selinexor in combination with venetoclax from the Clinical Trial Registry website. The first one is a combination of selinexor and venetoclax with cytarabine and fludarabine for children or young adults with R/R AML. The second one is untreated AML who are ineligible for intensive chemotherapy but MRD positive after azacitidine and venetoclax, selinexor 60 mg on D15 and D22 will be added. The third one is the use of only selinexor in combination with venetoclax for naïve and refractory AML. However, no results of the three trials were posted. To our knowledge, there are no prior published studies of selinexor combined with venetoclax in AML or myeloid sarcoma patients.

In our study, six of our patients were treated with regimens including selinexor and venetoclax. Of the six patients, three achieved CR, two achieved PR, and the ORR was 83.3%. Two patients with monocytic leukemia, AML-M4, achieved CR after treatment containing selinexor and venetoclax. In addition, another patient with AML-M5 achieved CR after treatment containing selinexor. Therefore, for patients with monocytic leukemia, venetoclax alone should be avoided as much as possible, and selinexor is superior to venetoclax. Of course, this is only our treatment experience based on a small number of cases, and further conclusions need to be verified by more cases.

The intensive chemotherapy approach is accompanied by several potential complications, including prolonged marrow aplasia (Carter et al., 2020). Patient No. 7 who underwent autologous hematopoietic stem cell transplantation (auto-HSCT) but had a relapse 8 months after transplantation did not have a suitable donor for allo-HSCT and could not tolerate intensive chemotherapy because of her poor marrow function. In addition, patient No. 10 also had marrow aplasia after previous intensive chemotherapy. The preclinical studies showed that selinexor has potent cytotoxic activity in AML cell lines and murine models with minimal effects on normal bone marrow (Ranganathan et al., 2012; Etchin et al., 2013; Etchin et al., 2016). For such patients, selinexor-containing chemotherapy-free or low-dose chemotherapy regimens are more suitable because it does not affect bone marrow function, which also is one of the advantages of this regimen.

Patient No. 1 was diagnosed with gastric myeloid sarcoma. Isolated Myeloid sarcoma is rare and incidence has been limited to case reports which often pose therapeutic dilemmas (Bakst et al., 2011). Given the inability of the patient to tolerate intensive chemotherapy for the lesion in the stomach and the efficacy of selinexor in AML as well as the tolerability of selinexor in patients with gastric cancer (Subhash et al., 2018), a low-dose chemotherapy regimen containing selinexor was used. After receiving two cycles of low-dose chemotherapy regimen, the SUV value of the gastric area went down from 12.1 to 7.1. Then another regimen containing selinexor brought the SUV value down to 2.1, the patient achieved a PR and then received allo-HSCT. Our study only included one patient with myeloid sarcoma, but it also provides supportive data for the treatment of myeloid sarcoma, especially in patients who are not suitable for chemotherapy.

R/R AML patients are mostly accompanied by adverse gene mutations, such as TP53, ASXL1, RUNX1, and so on (Döhner et al., 2022). In our study, patients with these adverse mutations achieved CR or PR after treatment containing selinexor. MLL-PTD often occurs in elderly patients and consists of 3%–5% of *de novo* AML, having a bad prognosis (Kihara et al., 2014). Both patient No. 2 and patient No. 5 had MLL-PTD mutation and had improved outcomes after receiving the selinexor-containing regimen. It is not clear which type of AML patients would benefit the most from a selinexor-containing regimen for the small patient cohort, which is the limitation of our study. The previous paper found biomarkers for personalized treatment of acute myeloid leukemia with doxorubicin as well as etoposide *in silico* analysis and *in vitro* experiment (Turk et al., 2020). This gives us a hint that follow-up studies could further identify the best benefit groups in this way.

Allo-HSCT remains the only curative treatment for R/R AML and myeloid sarcoma (Tallman et al., 2019). And allo-HSCT is best performed in CR, which is preferred by allowing enough time for a robust graft-versus-leukemia (GVL) effect to be established and results may be even better in patients with a deeper remission documented by negative cytogenetic, molecular, or flow cytometric analyses (Rashidi et al., 2018). In contrast, allo-HSCT in patients with active leukemia at the time of transplant have poor outcomes with long-term survival rates of only about 20% (Jabbour et al., 2014; Othus et al., 2015). In our study, four patients received an allo-HSCT after achieving CR, one patient received an allo-HSCT after achieving PR and our transplantation rate was 50.0%, which was similar to that seen with selinexor in combination with standard and intensive chemotherapy regimens (Fiedler et al., 2020; Martinez Sanchez et al., 2021).

As for hematological toxicity, in terms of neutrophil and platelet recovery time, the regimen that selinexor combined with CLAG were 28 and 37 days respectively (Abboud et al., 2020), and the regimen that selinexor plus FLAG-Ida were 40 days and 21 days respectively (Martinez Sanchez et al., 2021). And the median time of neutrophil recovery was 35 days (range, 21–47) in selinexor plus fludarabine and cytarabine (Alexander et al., 2016). In our study, the median duration of grade 3/4 neutropenia and grade 3/4 thrombocytopenia were 7 days and 4 days respectively, which was shorter than that of selinexor combined with standard or intensive chemotherapy regimens. This reduces the incidence of serious infections and bleeding during periods of neutropenia and thrombocytopenia and increases the opportunity for transplantation.

In terms of nonhematologic AEs, all patients had grade 1–2 hyponatremia, with no grade 3–4 hyponatremia reported which is lower than the data reported in the literature (Alexander et al., 2016; Bhatnagar et al., 2020). Reversible cerebellar toxicity has been reported with selinexor at 70 mg/m^2^ in R/R AML patients (Alexander et al., 2016) and with selinexor at a dose of 85 mg/m^2^ in a patient with advanced solid tumors (Abdul Razak et al., 2016). One of the patients in this study had an acute cerebral infarction after receiving selinexor at 35 mg/m^2^, but it was judged to be unrelated to selinexor as the patient had a long history of venous thrombosis in the lower extremities, and symptoms resolved with treatment. One patient who received 10 cycles of chemotherapy regimens prior, suffered from septic shock during the treatment. No serious AEs were observed in the remaining patients during treatment, and the treatment was generally well tolerated. Mild AEs are also one of the advantages of our study, which allows patients to tolerate treatment without interruption due to adverse reactions. At the same time, the patients were in better PS and eligible patients could bridge to transplantation.

We have to admit that our study has limitations due to real-world study. The small patient cohort, the heterogeneity in treatment approach, lack of control group and short follow-up period are limitations of our study. However, given the lack of effective treatments for R/R AML and myeloid sarcoma, our current study could still provide useful information to hematologists. In the future, randomized controlled trials may provide stronger evidence of the efficacy and safety of the treatment.

In summary, in this study, chemotherapy-free or low-dose chemotherapy regimens with selinexor achieved good outcomes and was well tolerated in patients with R/R AML, providing the opportunity for transplantation, which could be a viable novel treatment option for patients with R/R AML.

## Data Availability

The original contributions presented in the study are included in the article/Supplementary Material, further inquiries can be directed to the corresponding author.

## References

[B1] Abboud R. Chendamarai E. Rettig M. P. Trinkaus K. M. Riedell P. A. Abboud C. N. (2020). Selinexor combined with cladribine, cytarabine, and filgrastim in relapsed or refractory acute myeloid leukemia. Haematologica 105 (8), e404–e407. 10.3324/haematol.2019.236810 31753931 PMC7395277

[B2] Abdul Razak A. R. Mau-Soerensen M. Gabrail N. Y. Gerecitano J. F. Shields A. F. Unger T. J. (2016). First-in-Class, first-in-human phase I study of selinexor, a selective inhibitor of nuclear export, in patients with advanced solid tumors. J. Clin. Oncol. 34 (34), 4142–4150. 10.1200/jco.2015.65.3949 26926685 PMC5562433

[B3] Alexander T. B. Lacayo N. J. Choi J. K. Ribeiro R. C. Pui C. H. Rubnitz J. E. (2016). Phase I study of selinexor, a selective inhibitor of nuclear export, in combination with fludarabine and cytarabine, in pediatric relapsed or refractory acute leukemia. J. Clin. Oncol. 34 (34), 4094–4101. 10.1200/JCO.2016.67.5066 27507877 PMC5477824

[B4] Bakst R. L. Tallman M. S. Douer D. Yahalom J. (2011). How I treat extramedullary acute myeloid leukemia. Blood 118 (14), 3785–3793. 10.1182/blood-2011-04-347229 21795742

[B5] Bhatnagar B. Zhao Q. Mims A. S. Vasu S. Behbehani G. K. Larkin K. (2020). Selinexor in combination with decitabine in patients with acute myeloid leukemia: Results from a phase 1 study. Leuk. Lymphoma 61 (2), 387–396. 10.1080/10428194.2019.1665664 31545113 PMC7552944

[B6] Bogenberger J. M. Kornblau S. M. Pierceall W. E. Lena R. Chow D. Shi C. X. (2014). BCL-2 family proteins as 5-Azacytidine-sensitizing targets and determinants of response in myeloid malignancies. Leukemia 28 (8), 1657–1665. 10.1038/leu.2014.44 24451410 PMC4131248

[B7] Carter J. L. Hege K. Yang J. Kalpage H. A. Su Y. Edwards H. (2020). Targeting multiple signaling pathways: The new approach to acute myeloid leukemia therapy. Signal Transduct. Target Ther. 5 (1), 288. 10.1038/s41392-020-00361-x 33335095 PMC7746731

[B8] Cree I. A. (2022). The WHO classification of haematolymphoid tumours. Leukemia 36 (7), 1701–1702. 10.1038/s41375-022-01625-x 35732830 PMC9252902

[B9] Das A. Wei G. Parikh K. Liu D. (2015). Selective inhibitors of nuclear export (SINE) in hematological malignancies. Exp. Hematol. Oncol. 4, 7. 10.1186/s40164-015-0002-5 25745591 PMC4350974

[B10] DeWolf S. Tallman M. S. (2020). How I treat relapsed or refractory AML. Blood 136 (9), 1023–1032. 10.1182/blood.2019001982 32518943 PMC7453152

[B11] DiNardo C. D. Maiti A. Rausch C. R. Pemmaraju N. Naqvi K. Daver N. G. (2020). 10-day decitabine with venetoclax for newly diagnosed intensive chemotherapy ineligible, and relapsed or refractory acute myeloid leukaemia: A single-centre, phase 2 trial. Lancet Haematol. 7 (10), e724–e736. 10.1016/s2352-3026(20)30210-6 32896301 PMC7549397

[B12] Döhner H. Wei A. H. Appelbaum F. R. Craddock C. DiNardo C. D. Dombret H. (2022). Diagnosis and management of AML in adults: 2022 recommendations from an international expert panel on behalf of the ELN. Blood 140 (12), 1345–1377. 10.1182/blood.2022016867 35797463

[B13] Etchin J. Montero J. Berezovskaya A. Le B. T. Kentsis A. Christie A. L. (2016). Activity of a selective inhibitor of nuclear export, selinexor (KPT-330), against AML-initiating cells engrafted into immunosuppressed NSG mice. Leukemia 30 (1), 190–199. 10.1038/leu.2015.194 26202935 PMC4994896

[B14] Etchin J. Sanda T. Mansour M. R. Kentsis A. Montero J. Le B. T. (2013). KPT-330 inhibitor of CRM1 (XPO1)-mediated nuclear export has selective anti-leukaemic activity in preclinical models of T-cell acute lymphoblastic leukaemia and acute myeloid leukaemia. Br. J. Haematol. 161 (1), 117–127. 10.1111/bjh.12231 23373539 PMC3980736

[B15] Ferrara F. Palmieri S. Mele G. (2004). Prognostic factors and therapeutic options for relapsed or refractory acute myeloid leukemia. Haematologica 89 (8), 998–1008. 15339685

[B16] Fiedler W. Chromik J. Amberg S. Kebenko M. Thol F. Schlipfenbacher V. (2020). A Phase II study of selinexor plus cytarabine and idarubicin in patients with relapsed/refractory acute myeloid leukaemia. Br. J. Haematol. 190 (3), e169–e173. 10.1111/bjh.16804 32515072

[B17] Fischer M. A. Friedlander S. Y. Arrate M. P. Chang H. Gorska A. E. Fuller L. D. (2020). Venetoclax response is enhanced by selective inhibitor of nuclear export compounds in hematologic malignancies. Blood Adv. 4 (3), 586–598. 10.1182/bloodadvances.2019000359 32045477 PMC7013257

[B18] Garzon R. Savona M. Baz R. Andreeff M. Gabrail N. Gutierrez M. (2017). A phase 1 clinical trial of single-agent selinexor in acute myeloid leukemia. Blood 129 (24), 3165–3174. 10.1182/blood-2016-11-750158 28336527 PMC5524530

[B19] Gebru M. T. Wang H. G. (2020). Therapeutic targeting of FLT3 and associated drug resistance in acute myeloid leukemia. J. Hematol. Oncol. 13 (1), 155. 10.1186/s13045-020-00992-1 33213500 PMC7678146

[B20] Heuser M. Freeman S. D. Ossenkoppele G. J. Buccisano F. Hourigan C. S. Ngai L. L. (2021). 2021 update on MRD in acute myeloid leukemia: A consensus document from the European LeukemiaNet MRD working party. Blood 138, 2753–2767. 10.1182/blood.2021013626 34724563 PMC8718623

[B21] Hing Z. A. Fung H. Y. Ranganathan P. Mitchell S. El-Gamal D. Woyach J. A. (2016). Next-generation XPO1 inhibitor shows improved efficacy and *in vivo* tolerability in hematological malignancies. Leukemia 30 (12), 2364–2372. 10.1038/leu.2016.136 27323910 PMC5143172

[B22] Ishizawa J. Kojima K. Hail N. Jr. Tabe Y. Andreeff M. (2015). Expression, function, and targeting of the nuclear exporter chromosome region maintenance 1 (CRM1) protein. Pharmacol. Ther. 153, 25–35. 10.1016/j.pharmthera.2015.06.001 26048327 PMC4526315

[B23] Isidori A. Ferrara F. (2021). The changing landscape for patients with relapsed/refractory acute myeloid leukaemia. Curr. Opin. Oncol. 33 (6), 635–641. 10.1097/cco.0000000000000780 34474436

[B24] Jabbour E. Daver N. Champlin R. Mathisen M. Oran B. Ciurea S. (2014). Allogeneic stem cell transplantation as initial salvage for patients with acute myeloid leukemia refractory to high-dose cytarabine-based induction chemotherapy. Am. J. Hematol. 89 (4), 395–398. 10.1002/ajh.23655 24375514 PMC4140180

[B25] Karol S. E. Alexander T. B. Budhraja A. Pounds S. B. Canavera K. Wang L. (2020). Venetoclax in combination with cytarabine with or without idarubicin in children with relapsed or refractory acute myeloid leukaemia: A phase 1, dose-escalation study. Lancet Oncol. 21 (4), 551–560. 10.1016/s1470-2045(20)30060-7 32171069 PMC7153631

[B26] Kayser S. Levis M. J. (2022). Updates on targeted therapies for acute myeloid leukaemia. Br. J. Haematol. 196 (2), 316–328. 10.1111/bjh.17746 34350585

[B27] Kihara R. Nagata Y. Kiyoi H. Kato T. Yamamoto E. Suzuki K. (2014). Comprehensive analysis of genetic alterations and their prognostic impacts in adult acute myeloid leukemia patients. Leukemia 28 (8), 1586–1595. 10.1038/leu.2014.55 24487413

[B28] Kojima K. Kornblau S. M. Ruvolo V. Dilip A. Duvvuri S. Davis R. E. (2013). Prognostic impact and targeting of CRM1 in acute myeloid leukemia. Blood 121 (20), 4166–4174. 10.1182/blood-2012-08-447581 23564911 PMC3656451

[B29] Lapalombella R. Sun Q. Williams K. Tangeman L. Jha S. Zhong Y. (2012). Selective inhibitors of nuclear export show that CRM1/XPO1 is a target in chronic lymphocytic leukemia. Blood 120 (23), 4621–4634. 10.1182/blood-2012-05-429506 23034282 PMC3512237

[B30] Luedtke D. A. Su Y. Liu S. Edwards H. Wang Y. Lin H. (2018). Inhibition of XPO1 enhances cell death induced by ABT-199 in acute myeloid leukaemia via Mcl-1. J. Cell Mol. Med. 22 (12), 6099–6111. 10.1111/jcmm.13886 30596398 PMC6237582

[B31] Ma J. Ge Z. (2021). Recent advances of targeted therapy in relapsed/refractory acute myeloid leukemia. Bosn. J. Basic Med. Sci. 21 (4), 409–421. 10.17305/bjbms.2020.5485 33577442 PMC8292864

[B32] Martinez Sanchez M. P. Megias-Vericat J. E. Rodriguez-Veiga R. Vives S. Bergua J. M. Torrent A. (2021). A phase I trial of selinexor plus FLAG-Ida for the treatment of refractory/relapsed adult acute myeloid leukemia patients. Ann. Hematol. 100 (6), 1497–1508. 10.1007/s00277-021-04542-8 33914097

[B33] Mims A. S. Blum W. (2019). Progress in the problem of relapsed or refractory acute myeloid leukemia. Curr. Opin. Hematol. 26 (2), 88–95. 10.1097/moh.0000000000000490 30640734

[B34] Niu X. Wang G. Wang Y. Caldwell J. T. Edwards H. Xie C. (2014). Acute myeloid leukemia cells harboring MLL fusion genes or with the acute promyelocytic leukemia phenotype are sensitive to the Bcl-2-selective inhibitor ABT-199. Leukemia 28 (7), 1557–1560. 10.1038/leu.2014.72 24531733 PMC4090260

[B35] Othus M. Appelbaum F. R. Petersdorf S. H. Kopecky K. J. Slovak M. Nevill T. (2015). Fate of patients with newly diagnosed acute myeloid leukemia who fail primary induction therapy. Biol. Blood Marrow Transpl. 21 (3), 559–564. 10.1016/j.bbmt.2014.10.025 PMC4386840 25536215

[B36] Pei S. Pollyea D. A. Gustafson A. Stevens B. M. Minhajuddin M. Fu R. (2020). Monocytic subclones confer resistance to venetoclax-based therapy in patients with acute myeloid leukemia. Cancer Discov. 10 (4), 536–551. 10.1158/2159-8290.Cd-19-0710 31974170 PMC7124979

[B37] Perl A. E. Hosono N. Montesinos P. Podoltsev N. Martinelli G. Panoskaltsis N. (2022). Clinical outcomes in patients with relapsed/refractory FLT3-mutated acute myeloid leukemia treated with gilteritinib who received prior midostaurin or sorafenib. Blood Cancer J. 12 (5), 84. 10.1038/s41408-022-00677-7 35637252 PMC9151663

[B38] Pollyea D. A. Pratz K. Letai A. Jonas B. A. Wei A. H. Pullarkat V. (2021). Venetoclax with azacitidine or decitabine in patients with newly diagnosed acute myeloid leukemia: Long term follow-up from a phase 1b study. Am. J. Hematol. 96 (2), 208–217. 10.1002/ajh.26039 33119898

[B39] Ranganathan P. Yu X. Na C. Santhanam R. Shacham S. Kauffman M. (2012). Preclinical activity of a novel CRM1 inhibitor in acute myeloid leukemia. Blood 120 (9), 1765–1773. 10.1182/blood-2012-04-423160 22677130 PMC3433086

[B40] Rashidi A. Weisdorf D. J. Bejanyan N. (2018). Treatment of relapsed/refractory acute myeloid leukaemia in adults. Br. J. Haematol. 181 (1), 27–37. 10.1111/bjh.15077 29318584

[B41] Röllig C. Serve H. Hüttmann A. Noppeney R. Müller-Tidow C. Krug U. (2015). Addition of sorafenib versus placebo to standard therapy in patients aged 60 years or younger with newly diagnosed acute myeloid leukaemia (SORAML): A multicentre, phase 2, randomised controlled trial. Lancet Oncol. 16 (16), 1691–1699. 10.1016/s1470-2045(15)00362-9 26549589

[B42] Senapedis W. T. Baloglu E. Landesman Y. (2014). Clinical translation of nuclear export inhibitors in cancer. Semin. Cancer Biol. 27, 74–86. 10.1016/j.semcancer.2014.04.005 24755012

[B43] Subhash V. V. Yeo M. S. Wang L. Tan S. H. Wong F. Y. Thuya W. L. (2018). Anti-tumor efficacy of Selinexor (KPT-330) in gastric cancer is dependent on nuclear accumulation of p53 tumor suppressor. Sci. Rep. 8 (1), 12248. 10.1038/s41598-018-30686-1 30115935 PMC6095850

[B44] Tallman M. S. Wang E. S. Altman J. K. Appelbaum F. R. Bhatt V. R. Bixby D. (2019). Acute myeloid leukemia, version 3.2019, NCCN clinical practice guidelines in Oncology. J. Natl. Compr. Canc Netw. 17 (6), 721–749. 10.6004/jnccn.2019.0028 31200351

[B45] Turk S. Turk C. Akbar M. W. Kucukkaraduman B. Isbilen M. Demirkol Canli S. (2020). Renin angiotensin system genes are biomarkers for personalized treatment of acute myeloid leukemia with Doxorubicin as well as etoposide. PLoS One 15 (11), e0242497. 10.1371/journal.pone.0242497 33237942 PMC7688131

[B46] Wang Y. Liu Q. F. Wu D. P. Wang J. B. Zhang X. Wang H. X. (2020). Impact of prophylactic/preemptive donor lymphocyte infusion and intensified conditioning for relapsed/refractory leukemia: A real-world study. Sci. China Life Sci. 63 (10), 1552–1564. 10.1007/s11427-019-1610-2 32086670

[B47] Wei A. H. Montesinos P. Ivanov V. DiNardo C. D. Novak J. Laribi K. (2020). Venetoclax plus LDAC for newly diagnosed AML ineligible for intensive chemotherapy: A phase 3 randomized placebo-controlled trial. Blood 135 (24), 2137–2145. 10.1182/blood.2020004856 32219442 PMC7290090

[B48] Wei A. H. Strickland S. A. Jr. Hou J. Z. Fiedler W. Lin T. L. Walter R. B. (2019). Venetoclax combined with low-dose cytarabine for previously untreated patients with acute myeloid leukemia: Results from a phase ib/II study. J. Clin. Oncol. 37 (15), 1277–1284. 10.1200/jco.18.01600 30892988 PMC6524989

[B49] Winters A. C. Gutman J. A. Purev E. Nakic M. Tobin J. Chase S. (2019). Real-world experience of venetoclax with azacitidine for untreated patients with acute myeloid leukemia. Blood Adv. 3 (20), 2911–2919. 10.1182/bloodadvances.2019000243 31648312 PMC6849960

[B50] Zhang Q. Riley-Gillis B. Han L. Jia Y. Lodi A. Zhang H. (2022). Activation of RAS/MAPK pathway confers MCL-1 mediated acquired resistance to BCL-2 inhibitor venetoclax in acute myeloid leukemia. Signal Transduct. Target Ther. 7 (1), 51. 10.1038/s41392-021-00870-3 35185150 PMC8858957

